# Blue–green water utilization in rice–fish cultivation towards sustainable food production

**DOI:** 10.1007/s13280-022-01711-5

**Published:** 2022-03-04

**Authors:** Nesar Ahmed, John Hornbuckle, Giovanni M. Turchini

**Affiliations:** 1 School of Life and Environmental Sciences, Deakin University, Burwood, VIC 3125 Australia; 2 Centre for Regional and Rural Futures, Deakin University, Griffith, Hanwood, NSW 2680 Australia

**Keywords:** Environmental sustainability, Food production, Integrated farming, Rainwater, Water efficiency

## Abstract

Integrated rice–fish culture is a competitive alternative to rice monoculture for environmental sustainability and food productivity. Compared to rice monoculture, rearing fish in rice field ecosystems could increase food (rice and fish) production from this coculture. Moreover, the water productivity of rice–fish coculture is considerably higher than that of rice monoculture, because of double cropping. Despite these benefits, rice–fish coculture has not yet been broadly practiced. One of the potential challenges for the wider adoption of rice–fish coculture is water management. There are two forms of water involved in rice–fish cultivation: (1) blue water–surface and groundwater, and (2) green water–soil water from rainfall. The aim of this article is to focus on key factors determining the adoption of rice–fish cultivation through the effective utilization of blue–green water. We suggest that the efficient application of blue and green water in rice–fish coculture could help confronting water scarcity, reducing water footprint, and increasing water productivity.

## Introduction

Integrated rice–fish culture has long been traditionally practiced in many Asian countries, including Bangladesh, China, India, Indonesia, Japan, Malaysia, Myanmar, the Philippines, Thailand, and Vietnam (Fernando 1993; Halwart and Gupta 2004; Hu et al. 2016). It has also been practiced, or at least trialed, in other continents of the world, including Africa, America (mainly South), Australia, and Europe (Halwart and Gupta 2004; Ofori et al. 2005; Halwart and van Dam 2006). A range of aquatic environmental conditions including irrigated, rainfed, and deepwater rice fields offer opportunities for fish culture (Rothuis et al. 1998; Mohanty et al. 2009; Mishra et al. 2014). Various aquatic species are usually grown in rice ecosystems, such as cyprinids,^1^ catfish, eels, milkfish, mullets, prawns, and tilapias (Halwart and Gupta 2004; Mishra and Mohanty 2004; Frei and Becker 2005a; Hu et al. 2016).

There are two categories of rice–fish systems: (1) capture, and (2) culture. In the capture system, wild fish enter rice ecosystems during heavy rains. In the culture system, farmers stock externally sourced fish in rice fields. Rice–fish culture can be again categorized into: (1) integrated, and (2) alternate. In the integrated system, fish and rice are grown concurrently, while both are produced rotationally in the alternate system. This article focuses on the integrated farming system.

Rice–fish coculture can be considered as an ecosystem-based approach because of the positive farming attributes through the complementary use of wetland, environment, and aquatic living resources (Frei and Becker 2005a; Ahmed and Garnett 2011). Positive environmental conditions in rice fields offer a broad range of ecosystem functions, including respiration, photosynthesis, water purification, and nutrient cycling (Halwart and Gupta 2004). Moreover, rice ecosystems provide benthic, planktonic, and periphytonic food for fish (Mustow 2002; Halwart and Gupta 2004). The consumption of aquatic plants by fish in rice fields reduces weed biomass, richness, and abundance by 59%, 62%, and 68%, respectively (Wan et al. 2019). Moreover, fish eat pests and insects in rice fields, and the reduction rate of planthoppers by fish is 26% (Xie et al. 2011), which is a strategy for integrated pest management (Horstkotte-Wesseler 1999; Berg and Tam 2018). Rice–fish coculture helps the conservation of aquatic biodiversity with maintaining ecosystems (Xie et al. 2011; Berg et al. 2012; Wan et al. 2019). Rice fields have beneficial impacts on fish biodiversity due to providing breeding–nursery–feeding habitats for some local species (Halwart and Gupta 2004; Halwart 2008). Ultimately, rice–fish coculture provides a broad range of ecosystem goods and services (Xie et al. 2011; Berg et al. 2012; Wan et al. 2019), and the ecosystem services value of rice–fish coculture is 38% greater than rice-only culture (Liu et al. 2020).

Because of environmental gains, rice–fish integration is a sustainable alternative to rice monoculture that is capable of delivering increased crop production. Rice–fish coculture is an effective practice of sustainable intensification, i.e., generating additional food from the equal area of land, with reduced ecological impacts (Godfray et al. 2010; Ahmed and Garnett 2011). Rice–fish farming is an ecological intensification towards multi-trophic food production (Wan et al. 2019). Rice–fish coculture add economic value to water and land through increasing crop production (Berg 2002; Ahmed et al. 2011). Compared to rice-only farming, rice–fish coculture provides 10% higher net income (Wan et al. 2019). Ultimately, rice–fish coculture increases crop productivity, economic profitability, and social sustainability (Lu and Li 2006; Ahmed and Garnett 2011; Xie et al. 2011; Bosma et al. 2012; Ahmed et al. 2014a; Saikia and Das 2015).

Despite environmental and socioeconomic advantages, rice–fish coculture has not yet been broadly practiced. Globally, 167 million ha of rice ecosystems exist (FAO 2020a), of which 134 million ha are appropriate for rice–fish coculture, however only 1% of rice ecosystems are under fish culture (Halwart and Gupta 2004). The potential is clearly immense, but a question arises that “why is the adoption of rice–fish coculture quite low?” The summary of this debate about the low adoption of rice–fish coculture including inadequate knowledge of farmers for both fish and rice production (Li 1988; Berg 2002), and widespread chemical application in intensive rice monoculture that cannot favor fish growth (Wilson and Tisdell 2001). Moreover, financial constraints as well as higher labor requirement for rice–fish coculture (Horstkotte-Wesseler 1999; Ahmed and Garnett 2011), low fish productivity (Gurung and Wagle 2005; Ahmed and Garnett 2011), technical and institutional constraints (Nabi 2008), and competition for water use (Nhan et al. 2007) were identified. One of the key bottlenecks and limiting factors for rice–fish coculture is water management, including drought, water scarcity, inadequate water depth, lack of irrigation facilities, and insufficient water control (Horstkotte-Wesseler 1999; Frei and Becker 2005a; Nabi 2008; Ahmed and Garnett 2011). Clearly, there is a research gap about water management in rice–fish culture. It is therefore necessary to focus on water management for the successful adoption of rice–fish coculture. Policy support in food and environmental sustainability with social responsibility must be needed for this integrated farming (Agovino et al. 2018; Scarpato et al. 2020).

Global freshwater withdrawal for crop production has been increasing considerably over the last few decades due to population growth, changing diet preference, and climate change (Falkenmark 2013; Lee and Bae 2015; UNESCO 2020). There is a high demand for freshwater in crop production, and irrigation is known to be responsible for the consumption of significant amounts of water (Brauman et al. 2013; UNESCO 2020). Competition for freshwater application between aquaculture and agriculture is growing rapidly (Pueppke et al. 2020). Reducing water usage whilst maximizing food production is crucial to address water scarcity and food insecurity (Molden 2007; Mancosu et al. 2015). Efficient water utilization for food production through rice–fish cultivation could address one or more sustainable development goals (SDG), such as SDG 2—zero hunger, SDG 12—responsible consumption and production, and SDG 14—life below water (FAO 2018 and 2020b). It is therefore proposed that rice–fish coculture can be adopted through the efficient application of available water resources to achieve the SDGs.

There are two categories of water^2^ associated for fish as well as crop production: (1) blue water—surface and groundwater, and (2) green water–rainwater absorbed by soil (Falkenmark and Rockström 2006; Ahmed et al. 2018). This article focuses on the application of blue and green water in rice–fish culture for sustainable food production. The aim of this article is to focus on vital factors deciding the adoption of rice–fish cultivation through the effective use of blue–green water. Finally, this article provides some recommendations for the efficient application of blue–green water in rice–fish culture towards sustainable food production.

## Rationale and methodology

The purpose of this review was that of assessing currently known limitations and constraints of water use in rice–fish cultivation, with a particular focus on blue and green water application. To achieve this, a systematic review process was developed and applied to assess the available literature on water use in rice–fish coculture.

The following keywords were initially selected for retrieving available literature: “rice”, “fish”, “culture”, “rice–fish farming”, “rice–fish cultivation”, “rice–fish coculture”, and “rice–fish integration”. Then the two following queries were implemented in the database Web of Science:

1. ‘rice–fish farming’ (all fields) or ‘rice–fish cultivation’ (all fields) or ‘rice–fish coculture’ (all fields) or ‘rice–fish integration’ (all fields) = 184 results,
2. ‘rice’ (all fields) and ‘fish’ (all fields) and ‘culture’ (all fields) = 719 results.

The two databases were combined, and in parallel, additional manual searching techniques such as scanning of reference lists and broad searches using Google Scholar, and targeted search in the websites of certain specialized organizations such as Food and Agriculture Organization of the United Nations (FAO), Network of Aquaculture Centres in Asia Pacific (NACA), and WorldFish were implemented to supplement the database and to retrieve more studies.

The following exclusion criteria were then applied for the first screening: duplications, articles published before 1990 (though some older key articles have been included where appropriate), articles published in any language other than English, commentaries, protocol papers, and short communications, were all excluded.

The remaining articles were then analyzed by assessing title and abstract, and all those that were deemed relevant for water use in rice–fish coculture, and blue and green water application in rice–fish cultivation were then selected and utilized in the present review. This systematic review applies narrative approach with an in-depth assessment of the selected articles. It also identifies literature gaps and reasons for more research is needed to fill them.

## Water for rice–fish culture

### Water requirement

**Fig. 1:**
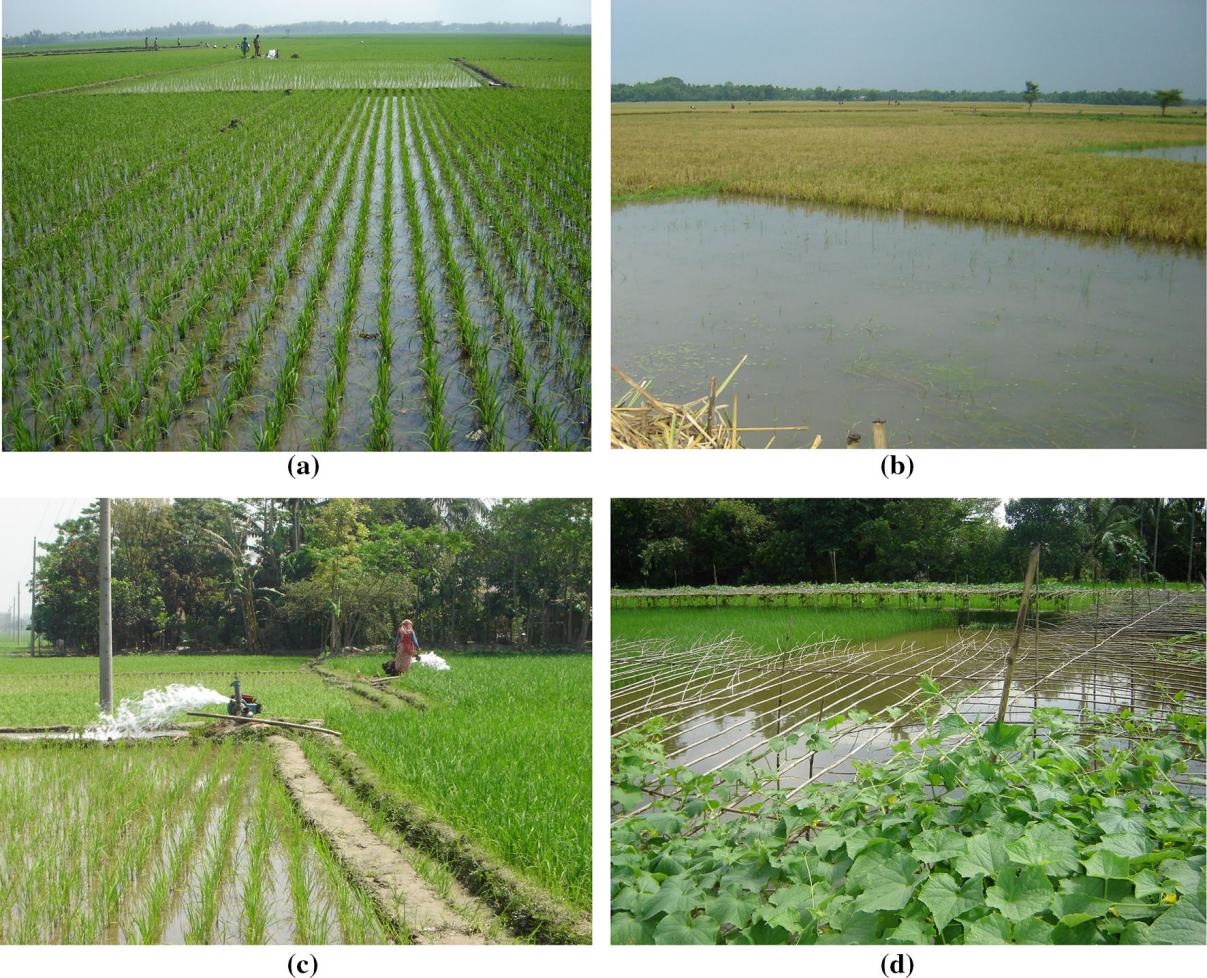
Rice fields constitute an important agro-ecological landscape, showing **a** continuous standing water in rice fields offers rice–fish coculture, **b** fish refuge contains more water depth to support fish culture, **c** irrigation facilities with proper drainage systems help rice–fish integration, and **d** higher dikes around rice fields with dike cropping protect fish escape during flood (photos by Nesar Ahmed)

Standing water^3^ in rice fields offers the best environment for the growth of rice. Although the water level in rice fields under traditional monoculture agronomic practice might vary from 2.5 to 20 cm, a continuous flooding of 2.5–7.5 cm water is considered the best environment for optimum rice yield in tropical conditions (Roger 1996; Horstkotte-Wesseler 1999). Thus, relatively shallow water is used in standard rice monoculture, and clearly more (deeper) water is required for rice–fish coculture. Fish require a sufficient level of water to survive, and thus, a greater depth with water quality must be maintained in rice ecosystems, where water exists for at least 4 months or longer. Many farmers prefer to have fish refuge,^4^ which is a deeper part of rice fields that support fish to grow successfully (Fig. 1). Fish refuges usually contain water depth of 50 cm below rice field level, and the favorable depth of water level is 65–70 cm for fish in refuges (Halwart and Gupta 2004). Water levels of about 30–40 cm in rice fields, and 100 cm in ditches, were maintained for prawn and small fish culture in Bangladesh (Wahab et al. 2008). Nevertheless, fish culture in rice fields requires 21 cm water depth (Sevilleja et al. 1992). Rice–fish coculture was carried out within 20 cm water depth in Bangladesh and China (Frei and Becker 2005b; Xie et al. 2011; Hu et al. 2013). In Vietnam, an average 27 cm of water level was reported for rice–fish culture (Berg 2002). Thus, it appears that practices may vary slightly in different regions.

The lack of access to sufficient water in rice fields is one of the vital limiting factors for the wider adoption of rice–fish coculture (Horstkotte-Wesseler 1999; Nabi 2008; Ahmed and Garnett 2011). Drought, irregular rainfall, and water shortage may seriously affect fish growth in rice fields. Fish become more stressed in low water depth that affect survival, growth, and reproduction performances (Portz et al. 2006). Low water levels in rice fields severely affect the total biomass of fish production (Khoa et al. 2005). However, low water depths are used to reduce water usage through seepage (Sudhir-Yadav et al. 2011; Carracelas et al. 2019). Therefore, rice–fish coculture is challenging when water supply is limited.

Contrary, too much water or flood is one of the key concerns for rice–fish coculture. Avoiding fish escape from rice ecosystems is challenging during inundation. Floods also permit wild and predatory fish entry to rice fields, which may reduce fish yield because of direct predation and disease transmission. Water quality in rice fields is also affected by floods due to contamination with land**-**based pollutants that undermine fish growth and production. In developing countries, small-scale farmers are unable to build their narrow and low dikes in rice fields due to financial constraint, and typical dikes are about 25–30 cm high with similar width (Halwart and Gupta 2004), making them vulnerable to flood. Typically, rice fields are not equipped with sluice gates that would allow effective management of water levels.

Since rice is the main crop in rice–fish coculture, fish rearing must adapt to the availability and requirement of water imposed for rice production. In Bangladesh and Vietnam, fish farming has been traditionally practiced in medium-flooded (50–150 cm) and deep-flooded (150–250 cm) rice fields (Dey et al. 2005). Creating greater dikes with netting and fencing around rice fields can help retain fish from escape during flood (Fig. 1).

### Crop and water productivity

In rice–fish integration, not only do fish represent an additional crop, but rice production itself is generally improved. The growing of fish in rice fields increases rice production by 8–20% (Table 1). The existence of fish in rice fields increases rice yields as the movement of fish help enhance dissolved O_2_ levels, recycling nutrients, increase soil organic matter, and control aquatic insects, plants, organic detritus, and plankton that compete with rice for energy and nutrients (Mustow 2002; Halwart and Gupta 2004; Giap et al. 2005; Ahmed and Garnett 2011; Xie et al. 2011).

**Table 1:** Productivity of rice and fish in integrated farming with increased rice yield compared to rice monoculture

Yield in rice monoculture (kg/ha)	Yield in rice-fish coculture (kg/ha)		Increased rice yield over rice monoculture (%)	Reference
	Rice	Fish		
3362	3629	980	8	Mishra and Mohanty (2004)
3370	3670	354	9	Gurung and Wagle (2005)
4702	5261	259	12	Ahmed and Garnett (2011)
5319	6382	345	20	Tsuruta et al. (2011)
Average 4188	Average 4736	Average 485	Average 12	This study

Rice–fish farming can be sustained when the trade-off between yields of rice and fish is balanced (Hu et al. 2016). The average annual yield of fish in rice–fish coculture is 485 kg/ha (Table 1), because of extensive aquaculture practices.^5^ Typical annual fish production in rice fields ranges from 200 to 700 kg/ha (Frei and Becker 2005a). Nonetheless, fish yield of up to 1027 kg/ha in rice ecosystems can be gained within four months under low stocking (15 000 fry/ha) farming systems (Mohanty et al. 2004). Fish yield of up to 1693 kg/ha can be attained within six months under rainfed conditions of rice–fish coculture (Mishra et al. 2014). Compared to rice-only farming, rice–fish coculture could increase 25% of more food (rice and fish) production (Table 2). It has been computed that the world fish production would increase by 1 million tons per year, if rice–fish culture would expand to only 10% of the currently irrigated rice fields, at fish production of 150 kg/ha/year (Halwart and Gupta 2004). With greater annual fish yield of 345 kg/ha in rice fields (Tsuruta et al. 2011), global annual fish production could increase by 4.69 million tons, if rice–fish culture extended to 10% of the world’s existing 134 million ha of potential rice ecosystems. Simultaneously, annual rice production itself would increase by 12.60 million tons, because of the presence of fish (Ahmed and Turchini 2021).

**Table 2:** Increased crop production in rice-fish coculture compared to rice monoculture

Statement	Production
Average rice production in rice monoculture	4188 kg/ha
Average rice production in rice-fish coculture	4736 kg/ha
Average increased rice yield in rice-fish coculture compared to rice monoculture	12%
Average fish production in rice-fish coculture	485 kg/ha
Total average crop (rice and fish) production in rice-fish coculture	5221 kg/ha
Crop production ratio in rice-fish coculture – rice: fish	91:9
Increased crop (rice and fish) production in rice-fish coculture compared to rice monoculture	1033 kg/ha
Increased crop (rice and fish) production in rice-fish coculture compared to rice monoculture	25%

*Source* Adapted from Table 1

One of the possible ways to increase water productivity is to enhance crop yield through rice–fish coculture. Water productivity is expressed as the net benefits per unit of water applied, which is the comparison of net return from crop yield to the volume of water used (Molden 2007). Water productivity can be assessed on physical and economic viewpoints, with: (1) physical water productivity (kg product/m^3^ water) being the proportion of culture output to the volume of water input, and (2) economic water productivity ($/m^3^ water) being the cash gained per unit of water consumed (Molden et al. 2010). The key approach to alleviate water scarcity is to increase agricultural water productivity. The ultimate purpose of enhancing water productivity is to increase food yield, income, and environmental benefits, without requiring a parallel increase in water usage (Molden et al. 2010). Water productivity can be improved through modernization of agricultural practices, water saving irrigation approaches, enhanced soil fertility, and increased yield (Dugan et al. 2006; Ali and Talukder 2008; Molden et al. 2010; Brauman et al. 2013). It can also be improved through integrated soil and water management (Falkenmark and Rockström 2006).

Increasing water productivity is an actual means of enhancing crop yield (Molden 2007; Brauman et al. 2013). Within this context, rice–fish cultivation can increase water productivity through the intensification and diversification of crops. Water productivity in coculture is usually greater than non**-**integrated farming systems. Ultimately, rice–fish integration is considered as being capable of delivery “more crop per drop” (Ahmed et al. 2014b). Water productivity can be increased through fish stocking in irrigation systems that is recognized as “integrated irrigated aquaculture” (Halwart and van Dam 2006). Water productivity in rice monoculture is highly inconsistent due to evapotranspiration and agronomic practices which affect total water input, and values varying from 0.15 to 1.60 kg/m^3^ (Molden et al. 2010; Steduto et al. 2012), on average 0.74 kg/m^3^ (Cai et al. 2010) and 1.09 kg/m^3^ (Zwart and Bastiaanssen 2004). Whereas water productivity in rice–fish coculture has been estimated at 1.21 kg/m^3^ (Ahmed et al. 2014b). Therefore, rice–fish integration can increase 11–64% physical water productivity.

Because of adding fish, which typically have higher monetary value compared to rice, the economic water productivity of rice–fish coculture (US$0.41/m^3^) is considerably higher than rice monoculture (US$0.19/m^3^) (Table 3). The economic water productivity of aquaculture is US$0.07–1.35/m^3^ (Molden et al. 2010). Adding high-value fish species in rice–fish coculture can have a favorable impact on the economic value of water productivity (Mohanty et al. 2009). Additionally, nutritional benefits from rice–fish coculture can be substantial due to adding fish. The nutrient water productivity of aquaculture (17–340 g of protein/m^3^ water) is the highest of all major food producing sectors, including animal and vegetable production (Molden et al. 2010). It is calculated that the nutrient water productivity of rice–fish coculture (97 g of protein/m^3^ water) is 87% higher than rice monoculture (52 g of protein/m^3^ water), even with a relatively low ratio (91:9) between rice and fish biomass (Table 3). This finding suggests that the nutrient water productivity of rice–fish coculture is over three times higher than that of beef (10–30 g of protein/m^3^ water) (Molden et al. 2010).

**Table 3:** A comparison of economic water productivity and nutrient water productivity between rice monoculture and rice-fish coculture

Feature	Farming system		Reference
	Rice	Rice-fish	
Physical crop production (kg/ha)	4188	5221 (Rice: fish = 91:9)	From Table 1
Water use (m^3^ water/kg)	1.32	0.83	Chapagain and Hoekstra (2011); Ahmed et al. (2014b)
Water productivity (kg/m^3^ water)	0.74	1.21	Cai et al. (2010); Ahmed et al. (2014b)
Farm-gate product value^a^ (US$/kg)	0.25	0.34 (Rice: $0.25/kg, fish: $1.17/kg)	Ahmed et al. (2011)
Economic water productivity^b^ (US$/m^3^ water)	0.19	0.41	This study
Protein level^c^ (g/kg product)	70	80 (Rice: 70 g, carp: 180 g)	Skibniewska et al. (2013), Saleh et al. (2019)
Nutrient water productivity^d^ (g of protein/m^3^ water)	52	97	This study

^a^In rice-fish coculture, the average farm-gate product value is calculated based on a ratio of rice and fish is 91:9. Current farm-gate price of rice and fish may considerably higher than the reference year

^b^Economic water productivity (US$/m^3^) = Farm-gate product value (US$/kg) × water productivity (kg/m^3^)

^c^In rice-fish coculture, nutrient water productivity is estimated based on a ratio of rice: fish is 91:9

^d^Nutrient water productivity (g of protein/m^3^ water) = Protein level (g/kg product) × water productivity (kg/m^3^)

## Linking blue and green water for rice–fish culture

### Blue water application

Blue water is categorized into freshwater, brackish water, and marine water; however, this study focuses on blue freshwater in the context of agronomic utilization. Blue water denotes groundwater as well as surface runoff in basins, canals, lakes, ponds, reservoirs, streams, and wetlands, which can be withdrawn^6^ for human utilization including irrigation (Falkenmark and Rockström 2006; Rockström et al. 2009; Hoff et al. 2010; Menzel and Matovelle 2010; Chapagain and Hoekstra 2011). Rainfall is separated into blue and green water, as rainwater that is not absorbed and retained by the soil, and then reaches in groundwater, is considered blue water (Sood et al. 2014). Globally, 39% of precipitation contributes to blue water of which 36% moves to the ocean (Molden 2007), and water in estuaries, seas, and oceans is blue water that supports coastal and marine ecosystems. Rainfall also permits blue water movement into farmlands (Falkenmark and Rockström 2006), and thus, water flow in rice fields is known as blue water.

Blue freshwater is universally applied in agriculture towards food production. About 70% of global blue water is consumed for irrigation (FAO 2016). Irrigated agriculture denotes 16% of total farmland but contributes to 36% of the world’s food production (Hansen 2015). Blue water scarcity has become an increasing concern due to the expansion of irrigated agriculture to boost crop yield to match the raising demand (Liu and Savenije 2008; Liu and Yang 2010). Reduced blue water availability for agronomic purposes can have dramatic negative impacts (Liu et al. 2018). It has been estimated that global rice production would be 39% lower if no irrigated blue water was used in cropland (Hoff et al. 2010).

The extension of crop production causes a deficit of blue water availability (Falkenmark and Rockström 2006), and land conversion to agriculture and blue water utilization by farmlands for food production are almost at their planetary limits (Sposito 2013). Increasing food production in the future will need to overcome the challenge of blue water scarcity (Liu and Savenije 2008; Liu and Yang 2010). It has been predicted that about 59% of the global human population will face blue water scarcity by 2050 (Rockström et al. 2009). It has been suggested that changing global cropping patterns may help minimize blue water scarcity (Chouchane et al. 2020).

**Fig. 2:**
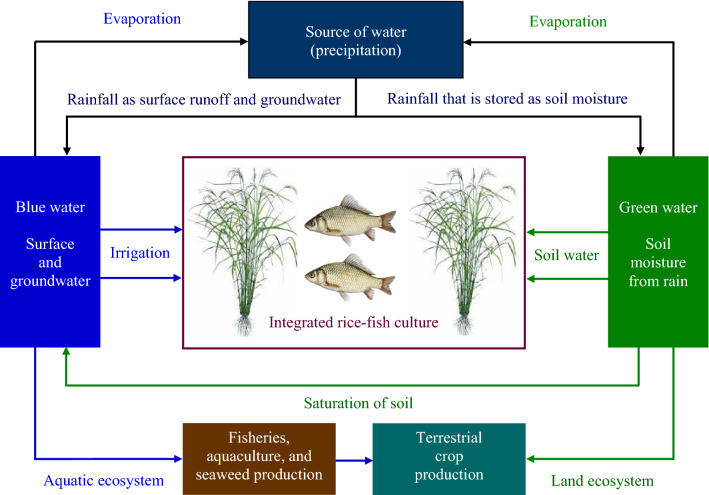
The source of blue and green water with their utilization in rice-fish cultivation and other purposes

Fish culture in rice fields intrinsically requires relatively more water, and it primarily depends on blue water (Table 4). Rearing fish in rice ecosystems is feasible relative to the availability of blue groundwater and surface runoff, but importantly, the application of blue water in rice–fish culture is also providing a significant contribution towards maintaining ecosystems (Fig. 2). Aquatic ecosystems completely rely on blue water that supports fish production and simultaneously conserves aquatic biodiversity (Molden 2007; Rockström et al. 2007).

**Table 4:** Water types with their application in rice-fish cultivation

Water type	Feature	Utilization in rice-fish cultivation
Blue water	Groundwater and surface runoff Water stored in lakes, ponds, and rivers (seas and oceans as well, though not usable for agronomic purposes) 39% of rainfall contributes to blue water	Precipitation allows blue water flow into rice fields Irrigated blue water can be utilized in rice-fish culture Blue water in rice-fish culture supports and maintains aquatic ecosystems
Green water	Rainfall stores as soil moisture Soil water generated by precipitation and utilized directly by plants 60% of rainfall contributes to green water	Rice fields receive green water indirectly, via soil moisture, from precipitation Rainfed rice fields support rice-fish coculture Green water provides environmental flow that supports rice-fish ecosystems

Rice–fish cultivation depends on irrigated blue water (Halwart and van Dam 2006; Ahmed et al. 2014b). Pumping blue groundwater, as well as diverted surface water, can be used for irrigation purposes and support rice–fish cultivation, and a variety of irrigation facilities with drainage systems can be applied. However, compared to traditional rice monoculture, higher irrigation costs are associated with rice–fish coculture, due to additional water requirement for fish (Rothuis et al. 1998). Nevertheless, despite increased irrigation costs, the efficient application of blue water in rice–fish culture can boost overall crop production, which in turn compensate production costs. Thus, it can be summarized that the rice–fish coculture approach, although “thirstier” than rice monoculture, is overall, more efficient in water utilization due to the dual use of blue water resource. The application of blue water in rice-fish farming increases water efficiency by providing dual crops, coupled with an increase yield of the main crop (rice) for the same water. Rice fields can access available blue water through canals and tributaries to support rice–fish culture. However, many rivers and tributaries are severely depleted worldwide because of using blue water for irrigation as well as other human activities. For example, the Indus and the Ganges River Basins jointly account for 25% of the blue water footprint associated with global food production during 1996–2005 (Mekonnen and Hoekstra 2011). In the Nile River Basin, over 5.4 million ha of agricultural land are using irrigation for crop production (Nile Basin Initiative 2020), but a reduction of blue water availability for irrigation purposes has grown at 0.3% per annum in the last two decades (Sulser et al. 2010). In the Mekong Delta, increased blue water withdrawal for agricultural production and hydropower could reduce water flow to serve levels that would impact food security in the region (Kirby et al. 2010; Boretti 2020). Therefore, effective utilization and maximizing the production outcomes of blue water use is critical moving forward and rice–fish culture has the ability to increase productivity and profitability per unit of blue water used to meet these challenges.

### Green water use

Green water represents precipitation that is deposited as soil moisture (Falkenmark and Rockström 2006; Rost et al. 2008; Chapagain and Hoekstra 2011). In other words, green water refers to soil water, which is originally derived by rainfall, that is absorbed and retained by soil and then utilized by terrestrial plants, through evapotranspiration for food production (Liu and Savenije 2008; Menzel and Matovelle 2010; Johansson et al. 2016). Green water is the portion of rainfall that infiltrates to become soil moisture or shortly stay on top of the soil and vegetation, then eventually returns to the atmosphere through evaporation and transpiration (Falkenmark and Rockström 2006; Rockström et al. 2009). Rainfall that does not move to a reservoir or aquifer turns into green water, which is the key source of water to generate food, feed, energy, fiber, and timber (Schyns et al. 2019). About 60% of rainfall contributes to green water (Molden 2007).

Most of the world’s crops are produced by using green water (Falkenmark and Rockström 2006; Rockström et al. 2007), and 85% of green water is directly utilized by terrestrial plants in global agriculture (Rost et al. 2008; Mekonnen and Hoekstra 2011). Opportunities for optimizing soil water accessibility and consumption by rainfed crops to boost production must be identified (Sposito 2013), as ultimately, the limited green water flow on the planet is utilized by nature and human society (Schyns et al. 2019). The amount of green water application in world food production is 4–5 times greater than the consumptive use of blue water (Hoff et al. 2010). Moreover, the future consumption of green water by the world’s food production, under predicted climate change and population growth scenarios, is expected to further increase to a higher scale than the direct consumptive blue water utilization (Rockström et al. 2009).

Rice fields obtain green water from rainfall which can perform a vital role in rice–fish cultivation (Table 4), as rainfed green water is appropriate for both rice and fish production (Fig. 2). Rainfed green water absorbed by soil or maintained in the ponded paddy helps rice growth by maintaining soil moisture (Chapagain and Hoekstra 2011). Rice plant roots consume soil moisture for transporting to the leaves, and thus, the growth of rice plant is a function of a green water availability and accessibility in the root zone (Falkenmark 2013). Increased amount of rainwater runoff (blue water) is generated when less water is absorbed by soil (green water), which will increase water runoff in rice fields to support fish culture. In tropical rainfed farming systems, a small portion of rainfall is accordingly used productively (Falkenmark and Rockström 2006). The proper utilization of green water is critical for obtaining higher yields from rice and fish production through integration, cultivation, and maintaining ecosystems. Ultimately, water runoff into rice fields is a positive contribution to the total ecological flow that helps aquatic species and environment (Bouman et al. 2006; Freed et al. 2020).

Rice fields can potentially keep rainwater, which might otherwise be misplaced from local environment, particularly via deposit to fish refuges, as well as adjacent ditches, that are commonly used for fish cultivation. In eastern India, the application of rainwater in low-lying rice ecosystems has been shown to help rice–fish coculture and supplement irrigation of horticultural crops (Mishra et al. 2014). Moreover, green water is significant for dike planting in surrounding rice fields as land ecosystems completely rely on green water (Rockström et al. 2007). In the Ganges River Basin, most consumptive water utilization for food production is green water (Sulser et al. 2010), and the largest green water footprint related to crop production was found for the Mississippi River Basin during 1996–2005 (Mekonnen and Hoekstra 2011).

## Blue–green water management

### Confronting water scarcity

Freshwater scarcity is a growing concern that may confront the adoption of rice–fish farming. Freshwater scarcity is expressed as the ratio of the total water footprint to the water availability during a specific period (Hoekstra et al. 2011). Water scarcity in food production arises not only from shortage of blue water, but also from lack of green water (Falkenmark 2013). Water shortage has intensified notably over the last decades, and presently one-third of the global population resides in areas reported to be experiencing blue and green water scarcity (Porkka et al. 2016).

**Fig. 3:**
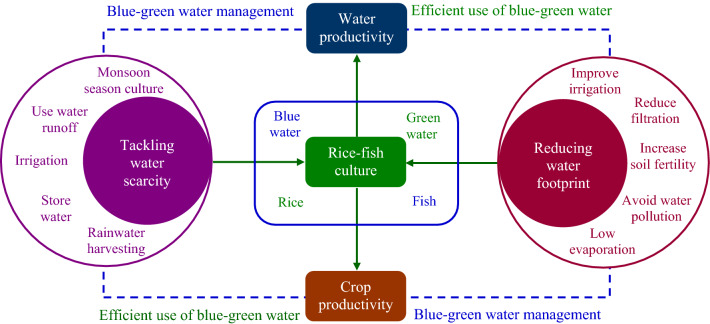
Tackling water scarcity and reducing water footprint in rice-fish coculture could increase water and crop productivity through the efficient use and management of blue–green water

Irrigated blue water and rainfall green water are important for rice–fish coculture to provide the require standing water and the maintenance of soil moisture in rice ecosystems. Rice and fish production relies on both blue and green water (Fig. 2). Since blue and green water are partitioned from rainfall, there are blue and green water trade-offs for food production (Karlberg et al. 2009). Differentiation between blue and green water consumption in a crop production situation is not straightforward (Hoekstra 2019), and ultimately, blue water cannot be isolated from green water in rice–fish culture as both water types are intimately interconnected and complementary to each other (Hansen 2015). Combined management of blue and green water is, therefore, needed for the effective utilization in rice–fish coculture. Increase in water productivity on irrigated farmland could decrease total water consumption by 8–15% in rainfall-limited regions (Brauman et al. 2013). Thus, the efficient utilization of blue–green water in rice–fish culture can offset for the lack of water, which in turn increase crop and water productivity (Fig. 3).

To address water scarcity in rice–fish cultivation, there are many options, including (1) rainy season cultivation, (2) use water runoff, (3) improve irrigation management, (4) store water in reservoirs, and (5) rainwater harvesting (Table 5). Fish culture in rice fields can be carried out in the rainy season to utilize the temporary high water level conditions. This seasonal cultivation reduces the irrigation demand of blue water for fish culture. Catching water runoff and storing in small reservoirs are effective blue water management strategies (Hoff et al. 2010; Wisser et al. 2010). These can increase the application of blue water resources for irrigation in rice–fish cultivation and are viable options to address water scarcity. Rice–fish cultivation through irrigation is one of the vital adaptation strategies to green water shortage in crop production. Green water is vital for irrigated farmland, when the source of blue water is scarce (Rost et al. 2008). The application of blue water irrigation can be coupled with available green water to support rice–fish culture. Improving irrigation systems including small-scale irrigation can be utilized to support the extra water requirement for fish cultivation (Speelman et al. 2008). Improving irrigation systems has a significant water savings potential, which increase water productivity by 9–15% (Jägermeyr et al. 2015). Additionally, by increasing profitability using a rice–fish production system, irrigation infrastructure can be upgraded to further improve water use efficiency in such systems.

**Table 5:** Blue-green water application in rice-fish cultivation for addressing water scarcity

Water type	Strategy	Effect on crop production	Effect on water resource
Blue water	Monsoon season cultivation, water store in reservoirs, and improving irrigation techniques to support extra water requirement in rice-fish culture	Increase rice production by area and by yield Increase fish production by area and by yield	Increase blue water consumptive use Increase water productivity
Green water	Rainwater collection, harvesting, capturing, storage, conservation, and supplementary irrigation can increase water flow in rice-fish ecosystems	Increase rice production by area and by yield Increase fish production by area and by yield	Increase green water consumptive use Increase water productivity

Green water scarcity is resulting from drought, irregular rainfall, rapid rainfall run-off, quick infiltration, dry soil, poor water holding capacity by soil, and climate change (Falkenmark 2013; Zisopoulou and Panagoulia 2021). Globally, rainwater harvesting is important for agricultural irrigation towards food production through storage and collection of rainfall during the rainy season (Wisser et al. 2010; Velasco-Muñoz et al. 2019). Accordingly, rainwater harvesting and subsequent conservation in rice fields could help rice–fish cultivation. In Africa and Asia, rainwater harvesting is an ancient idea for water management (Oweis and Hachum 2006; Liu et al. 2009), which is gaining significance for enhancing green water efficiency (AFED 2010). Precipitation storage in basins and reservoirs with supplementary irrigation of farmland has performed an important role in boosting crop yield (Gunnell and Krishnamurthy 2003; Wisser et al. 2010; Terêncio et al. 2018). The efficient use of rainwater harvesting can increase crop production by 10–20% (Hoff et al. 2010). The ancient idea of rainwater accumulation in ditches and small basins with additional irrigation of farmland can potentially intensify green water runoff, and thus, boost crop production by 35% in low-yield regions (Wisser et al. 2010). In the Amazon Basin, rainwater harvesting could be utilized for supplemental irrigation in secondary crops (e.g., cotton and maize) during the dry season (Lathuillière et al. 2016). Similarly, using the same conceptual approach, capturing more rainwater and storage in fish refuges can help rice–fish cultivation. Rainwater conservation in rice fields with refuges is paramount to support rice–fish culture (Mishra and Mohanty 2004).

### Reduce water footprint

The water footprint is a commonly used indicator of direct and indirect water consumption (Lovarelli et al. 2016; Pellicer-Martinez and Martinez-Paz 2016). In crop production, the water footprint is computed as volume of water relative to mass produced (m^3^ water/kg product), which is the opposite of water productivity (kg product/m^3^ water) (Mekonnen and Hoekstra 2014). During the period 1996–2005, the global annual average water footprint of humanity to be partitioned as 74% green, 15% grey, and 11% blue water, of which agriculture contributes 92% (Hoekstra and Mekonnen 2012). The global average water footprint of rice monoculture is 1.32 m^3^/kg, of which 0.58 m^3^/kg (44%) blue, 0.63 m^3^/kg (48%) green, and 0.11 m^3^/kg (8%) grey water (Chapagain and Hoekstra 2011). The water footprint of rice production is important in South Asian countries, where aquaculture is widespread. Water footprint reduction in irrigated blue water food production systems is important for improving overall sustainability and viability of the sector. Thus, a more efficient water resource utilization through appropriate irrigation techniques and other strategies should be considered to reduce blue water footprint (Chukalla et al. 2015).

Rice–fish integration is measured to be a water**-**efficient culture system due to non**-**consumptive and non**-**depletive water utilization by fish, as water is still being available for other practices (Dugan et al. 2006; Halwart and van Dam 2006; Verdegem et al. 2006; Abdul-Rahman et al. 2011). Thus, adding fish in rice fields will not significantly increase water footprint (apart from small increases due to increased water depths). On the other hand, it can significantly increase water productivity and profitability, considering rice and fish biomass. In practice, water use for rice production and that for rice–fish production is the same, and thus, transforming rice monoculture to rice–fish coculture is possible without extra water footprint. Nevertheless, analyzing the water footprint of rice–fish coculture in finer details, there is increased water footprint directly generated by aquaculture practices, but also there are significant production benefits originating by coupling rice and fish culture. The water footprint of aquaculture is due to a combination of water lost and water pollution. Water losses in rice–fish culture are similar to aquaculture practices in ponds. Water losses in fish farms are related to infiltration (i.e., seepage) and evaporation. Water losses from freshwater ponds through infiltration and evaporation are 5.5 and 2.7–6.3 mm/day, respectively (Verdegem and Bosma 2009). Similarly, water losses from rice fields through infiltration and evapotranspiration are 7.4 and 4–7 mm/day, respectively (Tomar and O’Toole 1980; Xu et al. 2018).

Water non-consumptive use for fish cultivation and its reuse for rice production can increase water productivity, which in turn minimizes water footprint in rice–fish ecosystems (Ahmed et al. 2014b). Fish can be integrated in rice fields, if sufficient water remains available, which ultimately increase crop and water productivity (Fig. 3). Moreover, water footprint in rice–fish culture can be further minimized through: (1) improving irrigation management, (2) reducing infiltration, (3) increasing soil fertility, (4) avoiding water pollution, and (5) minimizing evaporation.

**Fig. 4:**
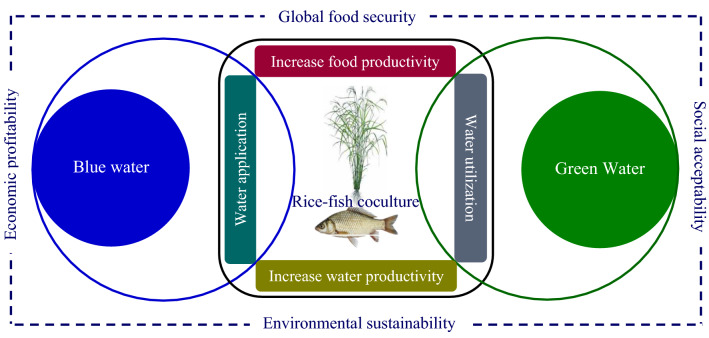
Rice-fish coculture maintains environmental sustainability for enhancing crop and water productivity, which may increase food security, economic profitability, and social acceptability

The presence of fish with their excrement in rice fields can increase nitrogen and phosphorus (Li 1988; Frei and Becker 2005a; Lin and Wu 2020). Swimming and foraging activities of fish in rice–fish coculture help nutrient recycling (Giap et al. 2005). Thus, fish increase soil fertility^7^ in rice fields, which can reduce fertilizer requirement. Moreover, fish consume insects and pests in rice ecosystems that reduce the application of insecticide and pesticide (Horstkotte-Wesseler 1999; Berg and Tam 2018). Compared to rice monoculture, rice–fish coculture requires 68% and 24% less use of pesticide and chemical fertilizer, respectively (Xie et al. 2011). Thus, grey water footprint, which is water pollution related to the use of fertilizer and pesticide, can be reduced through rice–fish culture (Chapagain and Hoekstra 2011; Mekonnen and Hoekstra 2011; Aldaya et al. 2020). The presence of fish as an important crop for farmers, and susceptibility of fish to chemical fertilizer and pesticide, would force farmers to be minimized any possible application of chemicals into rice fields. This in turn maintains water quality to increase rice and fish productivity that will greatly reduce grey water footprint. Additionally, rice–fish coculture can also keep water cleaner by absorbing nutrients with reducing organic pollutants. Moreover, higher growth of rice plants can offer more shelter and shade for fish during the summer period to decrease thermal stress (Lin and Wu 2020), which in turn may reduce water evaporation. Ultimately, rice–fish coculture maintains environmental sustainability for crop and water productivity to increase food security, economic profitability, and social acceptability (Fig. 4).

## Conclusions

Integrated rice–fish cultivation has the potential to increase net food (rice and fish) production, while simultaneously reducing water footprint, compared to traditionally practiced rice monoculture. However, despite increased food production with various environmental benefits, rice–fish coculture has not yet been broadly practiced. One of the potential challenges for the greater adoption of rice–fish coculture is water scarcity. Rice–fish coculture requires more water than rice monoculture. Hence, access to water is important for rice–fish coculture due to require extra water for the habitat of fish in rice fields.

The effective application of blue–green water in rice–fish coculture can improve water efficiency by delivering dual production benefits with increase yields. Fish farming in rice fields primarily relies on the availability of blue groundwater and surface runoff. Rice fields also receive soil moisture from green water precipitation. Irrigated blue water and rainfall green water are significant for rice–fish coculture due to maintain standing water and soil moisture in rice fields to increase crop and water productivity. Consequently, the water productivity in rice–fish coculture is considerably higher than rice monoculture.

The appropriate management policy of blue–green water is required for the effective utilization in rice–fish cultivation. Blue water irrigation with green water rainfall can help resist water scarcity for rice–fish coculture. The efficient application of blue–green water with specific management strategies in rice–fish cultivation could help in reducing water footprint. Management strategies, including improving irrigation systems and rainwater harvesting could address water scarcity. Ultimately, the efficient application of blue–green water in rice–fish culture can increase crop and water productivity. Eventually, the effective application of blue–green water is a viable tool towards addressing water scarcity and the increase of the adoption of rice–fish cultivation would ultimately contribute towards reducing water footprint. Key stakeholders including farming communities, government and non**-**governmental organizations, international agencies, policymakers, and researchers need to collaborate to support blue–green water management for the wider adoption of rice–fish coculture. Despite comprehensive review of this manuscript, there are research limitations for policy formulation in terms of blue–green water utilization in rice–fish cultivation. Thus, empirical research with particular focus on agronomic and aquaculture practices, as well as bioeconomic and ecosystem approaches, is required to recognize the effective application potentials of blue–green water in rice–fish culture, for a wider adoption of this food production system towards a sustainable food production globally.

## Footnotes

1 The choice of fish species is vital, as common carp (*Cyprinus carpio*) in rice fields can destroy freshly rooted rice seedlings by eating and uproot them (Halwart and Gupta 2004).

2 Besides blue and green water, two other forms of water are involved in the environment: (1) polluted grey water, and (2) white water—evaporated rainwater returns to the atmosphere (Savenije 2000; Chapagain and Hoekstra 2011).

3 A move from permanent flooding to intermittent irrigation as well as non-flooded conditions may favor for the “System of Rice Intensification (SRI)”, which uses less water than conventional farming (Dobermann 2004; Thakur et al. 2014). However, fish farming in rice fields requires continuous standing water.

4 There are various types of refuges in rice fields, including depressions or trenches. A fish refuge also acts to facilitate fish catch at the end of rice harvest.

5 Intensive aquaculture practices in rice-fish coculture are not viable due to shallow water level and short period of rice culture.

6 Water withdrawal denotes water pumped from ground or surface water for human utilization. Some withdrawal water can be restored and reused to the environment. Non-returned water refers to consumptive water that is incorporated into organisms or products, evaporated, or either way lost.

7 Soil fertility in rice-fish culture can increase crop and water productivity by preventing water seepage and nutrient loss through enhancing organic and physical matter that influence soil structure.
